# Comparative characterization and osteogenic / adipogenic differentiation of mesenchymal stem cells derived from male rat hair follicles and bone marrow

**DOI:** 10.1186/s13619-020-00051-7

**Published:** 2020-08-11

**Authors:** Abdel Kader A. Zaki, Tariq I. Almundarij, Faten A. M. Abo-Aziza

**Affiliations:** 1 Department of Veterinary Medicine, College of Agriculture and Veterinary Medicine, Qassim University, Buraydah, Saudi Arabia; 2 Department of Physiology, Faculty of Veterinary Medicine, Cairo University, Giza, Egypt; 3 Department of Parasitology and Animal Diseases, Veterinary Research Division, National Research Centre, Cairo, Egypt

**Keywords:** Stem cell, Bone marrow, Hair follicle, Osteogenesis, Adipogenesis

## Abstract

Clinical applications of cell therapy and tissue regeneration under different conditions need a multiplicity of adult stem cell sources. Up to date, little is available on the comparative isolation, characterization, proliferation, rapid amplification, and osteogenic/adipogenic differentiation of rat mesenchymal stem cells (MSCs) isolated from living bulge cells of the hair follicle (HF) and bone marrow (BM) from the same animal. This work hopes to use HF-MSCs as an additional adult stem cell source for research and application. After reaching 80% confluence, the cell counting, viability %, and yields of HF-MSCs and BM-MSCs were nearly similar. The viability % was 91.41 ± 2.98 and 93.11 ± 3.06 while the cells yield of initial seeding was 33.15 ± 2.76 and 34.22 ± 3.99 and of second passage was 28.76 ± 1.01 and 29.56 ± 3.11 for HF-MSCs and BM-MSCs respectively. Clusters of differentiation (CDs) analysis revealed that HF-MSCs were positively expressed CD34, CD73 and CD200 and negatively expressed CD45. BM-MSCs were positively expressed CD73 and CD200 and negatively expressed of CD34 and CD45. The proliferation of HF-MSCs and BM-MSCs was determined by means of incorporation of Brd-U, population doubling time (PDT) assays and the quantity of formazan release. The percentage of Brd-U positive cells and PDT were relatively similar in both types of cells. The proliferation, as expressed by the quantity of formazan assay in confluent cells, revealed that the quantity of release by BM-MSCs was slightly higher than HF-MSCs. Adipogenic differentiated BM-MSCs showed moderate accumulation of oil red-O stained lipid droplets when compared to that of HF-MSCs which exhibited high stain. The total lipid concentration was significantly higher in adipogenic differentiated HF-MSCs than BM-MSCs (*P* < 0.05). It was found that activity of bone alkaline phosphatase and calcium concentration were significantly higher (*P* < 0.01 and *P* < 0.05 respectively) in osteogenic differentiated BM-MSCs than that of HF-MSCs. The present findings demonstrate that the HF-MSCs are very similar in most tested characteristics to BM-MSCs with the exception of differentiation. Additionally; no issues have been reported during the collection of HF-MSCs. Therefore, the HF may represent a suitable and accessible source for adult stem cells and can be considered an ideal cell source for adipogenesis research.

## Background

Mesenchymal stem cells (MSCs) are cells having the capacity to survive in vivo and in vitro for a long time with self-renewal and differentiation capabilities to numerous cell lineages (Fortier and Travis 2011). These cells are responsible for tissues and organs regeneration and immunomodulation (Abo-Aziza and Zaki 2018). MSCs have been obtained from approximately all adult tissue types as whole umbilical cord (Zhang et al. 2017), umbilical cord blood (Abo-Aziza et al. 2018)**,** Wharton’s jelly (Hammam et al. 2016), bone marrow (Abo-Aziza et al. 2019a; Abo-Aziza et al. 2019b)**,** placenta, amniotic fluid (Park et al. 2012) and adipose tissue (Lee et al. 2015). Each source has been reported to vary in its proliferative and multilineage potential (Brevini and Gandolfi 2013)**.** In bone marrow (BM), MSCs represent a minimal part of nucleated cells. Despite this limited number, these cells can be purified after adhesion in plates and expanded easily. The adherent cells are usually cultured in suitable media and basic growth factors. The MSCs are expanded rapidly to reach required confluence at two to 3 weeks (Al-Mutairi et al. 2019). MSCs can be expanded and passaged through seeding limited number of cells in culture plates or flasks. MSCs can be differentiated into many cell types according to the types of differentiation media. Moreover, there remains a great interest in BM as the main source of MSCs for many preclinical and clinical investigations (Mori et al. 2017)**.**

Many advantages and disadvantages of BM origin of MSCs were described (Oryan et al. 2017). The advantages are the high culturing stability, reachable source for cell harvesting, easily prepared and high differentiation capacity. However, the disadvantages are the required euthanization, painful harvesting process, lowest proliferation capacity and risk of infection with bone tissue. Although some adult sources are relatively hard to be used as a tissue source for MSCs isolation, hair follicles (HFs) are newly and easily attainable. Beside the function of skin as a thermoregulatory, protective and conservative organ, it harbors many stem cells. The skin has two distinctive layers with many well defined histological structures like HFs and sebaceous glands (SG). HF is a complicated mini organ and undergoes active growing (anagen), regression (catagen) and resting (telogen) cyclical stages (Schneider et al. 2009). It made up of shaft with outer and inner root sheaths. HF has a microenvironment niche or “bulge” for mature stem cells called hair follicle stem cells (HF-MSCs). Bulge is located near the SG and the contractile hair muscle (Inoue et al. 2009). Bulge region was previously named “Haarbett” (hair bed) or “Wulst” (bulge or convexity) (Cotsarelis 2006). HF-MSCs were remained in a form of latency and triggered by pathological conditions or changes of normal conditions like tissue damage and wound repair throughout the period of living of organism (Voog and Jones 2010). The bulge region is composed of three distinct compartments: the lower part creates the inner cells coating the HF and constantly remodels during the hair’s cycle, the upper part is self-regenerating and is permanent (Guasch 2017) and the isthmus region that contains stem cells participated the formation of interfollicular epidermis and SGs especially during each anagen phase (Rompolas and Greco 2014). HF-MSCs are also situated inside the external sheath of the opening of the isthmus (Purba et al. 2014). HF-MSCs are self-regenerating and exhibit a broad potential to differentiate into multi lineages under appropriate conditions (Turksen 2015). HF-MSCs can not only differentiate into HFs, but it may also have the capacity to differentiate into SG, sweat glands, keratinocytes and inter-follicular epidermis (IFE) (Gentile et al. 2017). HF-MSCs can move down the hair shaft where they differentiated to internal HF (Turksen 2015). Special type of HF-MSCs was recently used to form cardiac muscle cells (Yamazaki et al. 2016; Shirai et al. 2017) and can fully repair the damaged sciatic nerve of mice (Obara et al. 2019) and cortical dysplasia (Omidi et al. 2015).

HF-MSCs can express a variety of surface antigens (CDs). For example, in mice, CD34 is firstly discovered in epidermis (Trempus et al. 2003) and used as a marker for bulge cells of mice but not in human (Cotsarelis 2006). This marker was considered as human bone marrow hematopoietic stem cell marker but not in the mice (Osawa et al. 1996).

Although BM-MSCs has been extensively considered as a main source of adult stem cells for therapy and application, other studies showed that it may be easily accessible to find additional source (Mistriotis and Andreadis 2013). Thus, more investigating about HF-MSCs may lead to their application in injuries and degeneration. However, a major problem facing the scientists is the lacking of effective and constant methods for isolation and identification of HF-MSCs. Optimization of isolation and characterization of living bulge cells is essential for clinical applications in regenerative medicine. Therefore, the aim of this study is to compare the HF-MSCs with BM-MSCs to evaluate their morphology, cell number, viability, cell yield, proliferation efficiency and capability for osteogenesis and adipogenesis.

## Methods

### Animals

The experiment on animals was obeyed to a reference of the guidelines of the Saudi Council for experimental animals and was accepted by the Committee of Animal Research Ethical of Qassim University. All rats were received a commercial formulated food to furnish the entire nutrient recommended for rats (NRC Nutrient Requirements of Laboratory Animals 1995). The animals were maintained under light dark cycles (08.00–16.00 h).

### Isolation of bulge regions and BM for purification of HF-MSCs and BM-MSCs

Twenty adult male Wistar rats (6–8 weeks old) were euthanized under sodium pentobarbital anesthesia. Rat’s heads were isolated and washed with ethyl alcohol and sterilized with betadine followed by rinsing with phosphate buffer saline (PBS) at least three times. The skin near the beards, cheeks, lip pads and whisker area containing vibrissae was dissected by ophthalmic scissors and cut into small parts (2 mm X 2 mm). The tissues were washed with PBS then rinsed with equal volumes of Dulbecco’s Modified Eagle’s Medium and Ham’s F12 medium (DMEM/F12) enriched with two types of antibiotics, one type of antifungal drug and fetal bovine serum 10% (FBS, Sigma-Aldrich, USA) to remove cell remnants completely. The pieces were transferred to DMEM/F12 supplemented with the same volume of dispase (0.1%) and collagenase type I (0.1%) for 3 h at 37 °C (Sigma-Aldrich, USA). The subcutaneous fat and connective tissue were removed with fine forceps under a dissecting microscope to expose the rows of vibrissae without damaging the dermal layer. The whisker follicle was lifted out after removal of connective tissue and the dermis around it. The two ends of HFs were removed, leaving the bulge. Hundred isolated bulges were cut into fine parts, plated into collagen type І coated dish before cultivation. Coating of 60 mm dishes were done at room temperature for 1 h by diluted collagen type І (0.5 ml, 50 ml diluent) then solution was replaced by DMEM/F12 media containing 10% FBS for 2 h (Quan et al. 2016). Pieces of bulges were placed in dishes and incubated in 37 °C and 5% CO_2_ incubator to allow stacking within 1day. Three to 4 days after adhesion, the cells started to leave the bulge to the plate bottom (Nobakht et al. 2010; Nobakht et al. 2011). After plating, the HF-MSCs were growing as floated spheres and they were isolated from adhered cells in bottom of the culture. The medium containing spheres were centrifuged for passage and the pellet was gently dissociated using 1 ml pipette tip. The culture plates and the cells obtained were incubated in an equal volume of trypsin (0.125%) and EDTA (0.02%) for 1 hr. at 37 °C. Distributed cells were then filtered with a 35 μm cell sieve to remove the agglutinated cells and hair shaft. The floated cells were incubated in other collagen type І coated plates for another week with changing the media two times a week (Nobakht et al. 2010).

Rat BM was harvested according to the protocol modified by Abo-Aziza and Zaki **(Abo-Aziza and Zaki**^2018^**)**. Skin, muscles and connective tissue were removed as much as possible from hind limbs. The limb above the hip and below the ankle was cut; the bone ends were maintained to ensure sterility of the BM. The knee joint was carefully broken apart and the remaining connective tissue from both ends of femur and tibia was stripped. Bones were placed in a dish and washed by transferring through PBS three times. Ends of each bone were snipped off with scissors (keeping as close to end as possible to extract more BM) and were gently placed in sterile PBS. Ten cubic centimeter syringe was filled with pre-warmed complete conditioned DMEM media (1.0 g/L glucose + 10% FBS) and the needle was attached to force media through bone shaft to extract all red marrow into 100 mm Petri dish containing 1 ml of heparin (2000 IU/0.2 ml). This step was repeated a few times to ensure all marrow were removed. Cell mixture was pipetted up and down a few times using a syringe to pull large marrow pieces through needle gauged 18 for further dissociation. The suspension was washed and centrifuged twice with PBS to remove tissue remnants. BM was then diluted with DMEM and an aliquot was stained with Giemsa stain after methanol fixation. This step was done to detect cellular compartments and status of BM. Diluted BM were slowly doubled with Ficoll-Paque (Sigma-Aldrich, USA) and centrifuged at 400 rpm for 15 min in floating centrifuge for separation of mononuclear cells (MNCs). BM-MNCs were collected into another centrifuge tube, and then washed twice with PBS. Isolation of MNCs from BM was also performed using same volume of sodium carbonate buffer solution (0.1%) for lyses of erythrocytes (Lee et al. 2015)**.** Particularly, 1 ml volume of BM was used on the same volume of buffer solution for separation of MNCs at low speed centrifugation for 20 min. MNCs were incubated overnight at 37 °C and 5% CO_2_ to adhere and non-adherent cells were removed with alpha minimum essential medium (α-MEM). The medium was supplemented with FBS (20%), L-glutamine (2 mM), 2- mercaptoethanol (55 μM), and two types of antibiotics. After that, the medium was changed twice weekly. Sub culturing was performed when adherent cells of first cell culture reached 80% confluence and it was named passage zero (P0). Serial passage numbers of 0.25 × 10^6^ cells were designated using trypsin-EDTA (0.25%). The following passages were named consequently. All the previous procedures were directed in an air filtered laminar flow safety cabinet and by using sterile instruments.

### Test of cell viability

Before culturing in osteogenic or adipogenic media, the MNCs, HF-MSCs (P3) and BM-MSCs (P3) were tested for viability by trypan blue stain. The cells were suspended at appropriate concentration of 1 × 10^5^/ml in α-MEM (Quan et al. 2016). The mean of three repeated measurements were used. Viable cells did not take blue color while dead cells got blue and quantified in a Neubauer chamber haemocytometer. Cell vitality was calculated as follow:

$$ {\displaystyle \begin{array}{c}\mathrm{Total}\ \mathrm{count}=\mathrm{cell}\ \mathrm{number}\ \left(16\ \mathrm{square}\right)\times \mathrm{dilution}\times {10}^4=\mathrm{count}\kern0.5em \mathrm{x}2\mathrm{x}{10}^5/\mathrm{ml}.\\ {}\mathrm{The}\ \mathrm{viability}\%=100\times \mathrm{number}\ \mathrm{of}\ \mathrm{viable}\ \mathrm{cells}/\mathrm{number}\ \mathrm{of}\ \mathrm{total}\ \mathrm{cells}\ \left(\mathrm{viable}+\mathrm{dead}\right).\end{array}} $$

### Cell yield

Once HF-MSCs and BM-MSCs became 80% confluence in the P1 and P2, cells were enzymatically detached as described and counted. Cells/cm^2^ were assessed by hemocytometer under microscope.

### Phenotyping using flow cytometry analysis

For phenotyping of HF-MSCs and BM-MSCs, CDs markers were detected (Yamaza et al. 2015). Briefly, 0.2 × 10^6^ cells were incubated and stained with phycoerythrin (PE) conjugated antibodies specific for rat CD34, CD45, CD73, and CD200 (BD Biosciences). Stained cells were examined by a FACS Calibur flow cytometer (BD Biosciences). Negative and positive populations of cells were included in all analysis.

### Estimation of proliferation capability

Estimation of the proliferation of HF-MSCs and BM-MSCs was measured in both primary cultures and subcultures. Cultures were monitored using inverted light microscope with digital camera for capturing images. Furthermore, the cells were tested for colony formation efficiency, population doubling time (PDT) and dimethylthiazol-diphenyltetrazolium bromide (MTT, Sigma-Aldrich, USA) assays to compare the proliferation of the cells.

#### Determination of clonal growth rates as minimum doubling time by colony forming unit (CFU) assay

To measure the colony formation efficiency of HF-MSCs and BM-MSCs, a suspension of 1 × 10^3^ cells from developed confluence (80%) in the P2/ml complete culture media was cultured in a 3.5 cm dishes and the cultures were observed under inverted microscope after 3 weeks. Each group of cells counting over 50 cells was considered as a colony **(Kentaro et al.**^2012^**)**.

#### PDT assay

PDT assay was performed to measure the proliferation capacity of HF-MSCs and BM-MSCs (Ren et al. 2015). A total of 25 × 10^3^ cells was seeded and detached using Trypsin / EDTA upon reaching 80% confluence followed by passage. The PDT for each cell was monitored in P4 - P5 and in P 9 - P10, respectively. PDT was calculated with the following formula:

$$ \mathrm{PDT}=1/\left[3.23\ \left(\log\ \mathrm{N}\mathrm{H}-\log\ \mathrm{N}\right)/\left(\mathrm{t}2-\mathrm{t}1\right)\right] $$

NH: Harvested cell number of 80% confluence, N: number of inoculated cells, t1: time at inoculation, t2 time between inoculation and harvesting.

#### MTT assay

It was done as an indirect method to determine the activity of mitochondrial enzymes. Cells were incubated with 0.2 mg MTT /ml appropriate media, for 1 hr. at 37 °C in 96-well plates to form formazan after reduction. The solution was then removed and solubilization of formazan was done by the addition of 0.04 N HCL in 1 ml isopropanol. Five minutes after shaking, the quantity of formazan was assayed calorimetrically at a wavelength of 570 nm (Guan et al. 2011)**.**

#### Bromodeoxyuridine (Brd-U) integration assay

The proliferation of required confluent HF-MSCs and BM-MSCs was assessed using the Brd-U integration assay **(Kentaro et al.**^2012^**)**. Cells (1 × 10^4^ /well) were inoculated on two-well chamber slides (Nunc, Denmark) for 3 days followed by incubation with diluted Brd-U solution (Invitrogen, USA) for 1 day. Cells were then stained with Brd-U kit (Invitrogen, USA). To determine HF-MSCs and BM-MSCs proliferation capacity, positive and total cell numbers were counted in five images. The proliferation was exposed as Brd-U positive cells percentage over the total nucleated cells.

### Adipogenic differentiation

For adipogenic differentiation induction, P3 cells were maintained with adipogenic differentiation media for 3 weeks. This media contained factors essential for adipogenesis as glucose (4.5 g/L), dexamethasone (1 μM), indomethacin (0.5 mM), 3-isobutyl-1-methylxanthine (0.5 mM) and insulin (10 μg/ml). The percent of factors was conducted as previously described (Lee et al. 2015). All materials used were purchased from Sigma-Aldrich, USA.

#### Oil red-O staining

Evaluation of adipogenic differentiation of HF-MSCs and BM-MSCs was done as previously described **[**39**]**. Oil red-O solution (0.5%) in isopropanol was prepared and filtered with 0.2 mm filter. Before staining, working solution was prepared from a mixture of 18 ml stock solution and 12 ml distilled water and left for 1 h at room temperature, and then it was filtered before use. Conflated cells were fixed in 96- well plates with paraformaldehyde (4%) in PBS for 20 min. Fixed cells were stained for 20 min and then rinsed twice with PBS. Cells were examined by inverted microscope. The dye inside the cells was then eluted by incubation with isopropanol for 15 min. At the end, 200 μl of the solution was pipetted into each well of the plates and the optical density was read using ELISA reader at 540 nm (Yadav et al. 2013).

#### Total lipids assay

Chloroform methanol solvents (2:1, v/v) extraction method was used according to Petkovic´ et al. (Petković et al. 2005). Briefly, 2 ml from solvent was added to cell pellets obtained by centrifugation at low speed. The mixture was agitated and incubated for 30 min followed by addition of 400 μl sodium chloride (0.9%). The mixture was then agitated and centrifuged at low speed. The lower layer was collected for the analysis by using sulfo-phospho-vanillin calorimetric kits (Sigma-Aldrich, USA).

### Osteogenic differentiation

The cultural condition of osteogenic differentiation was used as established previously (Abo-Aziza et al. 2019a)**.** To provide osteogenic conditions, the culture medium of the confluent cultures of P3 was substituted with osteogenic medium for 3 weeks and consisting of α-MEM with FBS (10%), L-ascorbic acid 2-phosphate (100 μM), L-glutamine (2 mM), dexamethasone (10 nM) and β-glycerophosphate (2 mM). The media was supplemented with penicillin (100 U/ml), streptomycin (100 μg/ml). The media were changed every 2 days. All materials used were purchased from Sigma-Aldrich, USA.

#### Evaluation of osteogenic differentiation by alizarin red staining

Alizarin red stain was used to evaluate the presence of calcified tissues and mineralization potentiality inside HF-MSCs and BM-MSCs differentiated cells. After rinsing cells with PBS, 10% buffered formalin was used to fix cells for 10 min. Rinsing of formalin was done using distilled water, followed by staining with freshly prepared Alizarin red solution (1%) for 20 min. The stain was discarded by washing with distilled water. Finally, keeping cells wet by 1 ml of distilled water was done. Detection of mineralization of all groups was performed during 3 weeks while the cells were maintained in the differentiation media and at the end. The mineralized nodules were observed and graphed using inverted microscope (Huang et al. 2009)**.**

#### Bone alkaline phosphatase (B-ALP) activity

B-ALP activity that indicates osteogenic differentiation was performed using previous method (Choi et al. 2005; Leskelä et al. 2006). Briefly, HF-MSCs and BM-MSCs were cultured separately in osteogenic media for 3 weeks, and then rinsed with PBS and trypsinised for elution. Cell lysis was done by freezing and thawing for 2–3 times. B-ALP assay kit (Abcam) was used by addition of 100 μl/well of p-nitrophenyl phosphate to produce p-nitrophenol. P-nitrophenol was then assayed at 405 nm and the results were expressed as units/mg protein which was determined (Lowry et al. 1951)**.**

#### Calcium deposition assay

The amount of calcium deposited in HF-MSCs- and BM-MSCs- derived osteogenic cells was indicated as previously described (Salasznyk et al. 2004). Briefly, fixed number of colonies from plates showing 80% confluence were washed twice with PBS and hydrochloric acid extracting solution were used (0.5 N). Calcium was extracted from cells by agitation for 5 h at 4 °C, followed by centrifugation at low speed. The supernatant was used for calcium determination by colorimetric assay kit at 575 nm (Sigma-Aldrich, USA). Standard solutions were prepared in parallel to express the reading as mg/well.

### Histopathology

To observe the structure of bulge region of rat follicle, the skins containing HFs were fixed with 4% neutral-buffered paraformaldehyde. Routine histological procedures were done to form paraffin blocks. Thin sections were stained with hematoxylin and eosin (H&E) stains (Carson 1990).

### Statistical analysis

Software of SPSS version 20 was used and both ANOVA and Tukey’s post-test were done. Data were stated as the mean ± standard error (SE).

## Results

### Morphology

**Fig. 1:**
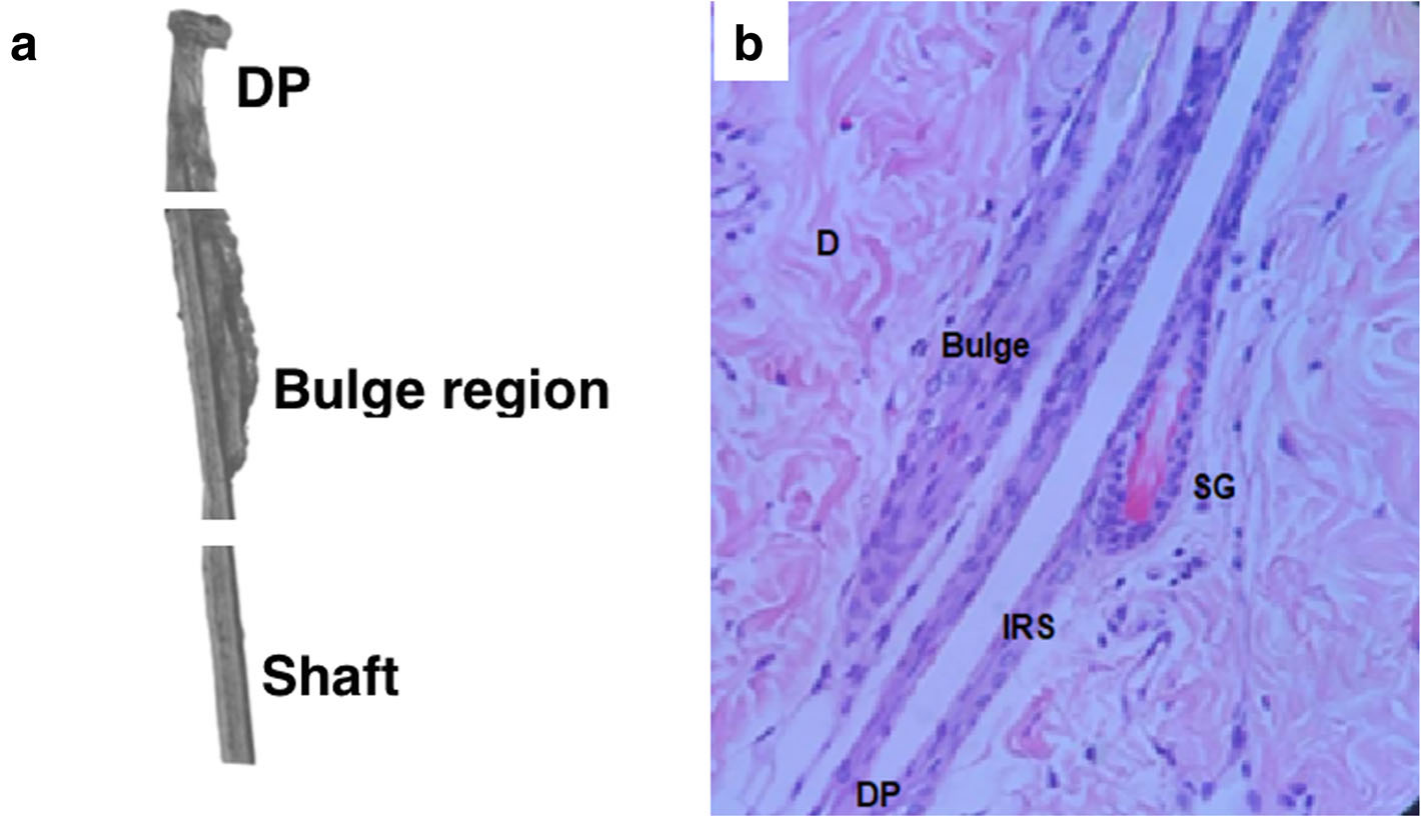
Hair follicle under dissecting microscope showed DP, bulge region and shaft (**a**). Histology of rat hair follicles stained with H and E, showing the bulge region, × 20 (**b**). Sebaceous glands: SG, inner root sheath: IRS and dermal papilla: DP

**Fig. 2:**
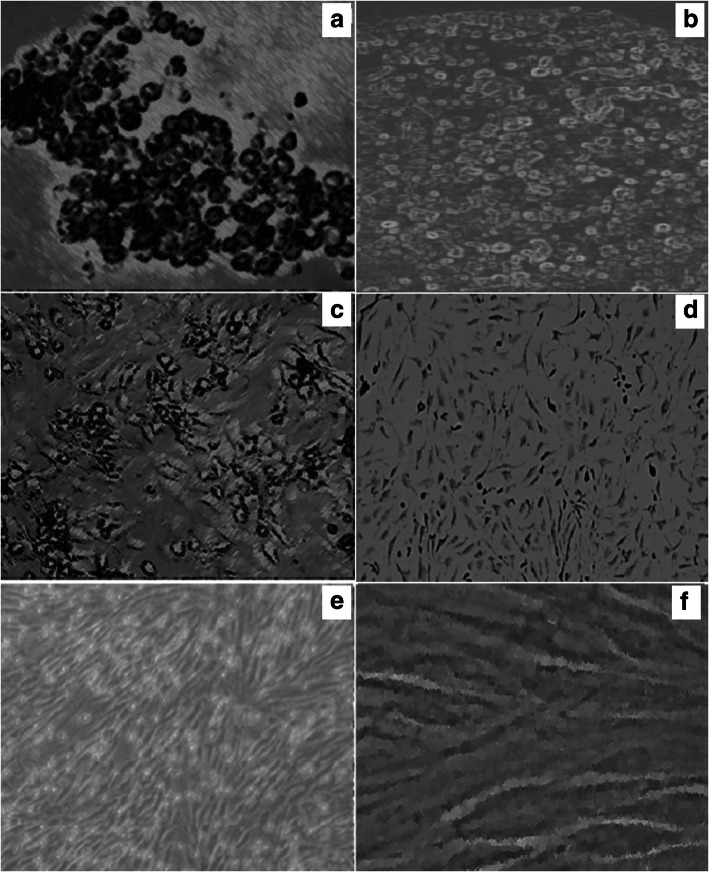
Photomicrograph under inverted microscope in the first day of cell culture of bulge region of dissected rat hair follicles after enzymatic dissolution. Most cells showed flagstone to cobblestone morphology, × 20 (**a**). Cells attached with epithelium or stratified appearance at the second day, × 20 (**b**). Fibroblastic appearance of cells appeared at the fourth day, × 20 (**c**). The cells were varied in morphology with a fibroblast-like cell type as the principal type, × 20 (**d**). Cultured HF-MSCs showing spindle shape and stellate shaped appearance and the cells reached high confluence within 2 weeks, (E, × 20 and F, × 40). HF-MSCs: hair follicle stem cells

HF of rat under dissecting microscope showed the region of interest (Fig. 1a). Histology of HF stained with H and E showed the bulge region, sebaceous glands (SG), inner root sheath (IRS) and dermal papilla (DP) (Fig. 1b). Isolated cells of the bulge regions of dissected rat HFs began to adhere after one to 2 days from culture. Most cells take epithelial, round, spindle, and flagstone to cobblestone appearance and were extensively adhered with plate under inverted microscope (Fig. 2a-b). The HF-MSCs appeared elongated and fibroblastic at subsequent 2 days of culture (Fig. 2c). After 1 week of culture, cell number was increased. Within another few days, the cells reached required confluency (80%) and were then passaged to another plate. After 10 days, most HF-MSCs (primary and then P2) showed typical morphology of spindle shaped stem cells and fibroblastic appearance with high colony-forming capacity (Fig. 2d). Cells reached over required confluence (100%) within 3 weeks (Fig. 2e-f).

**Fig. 3:**
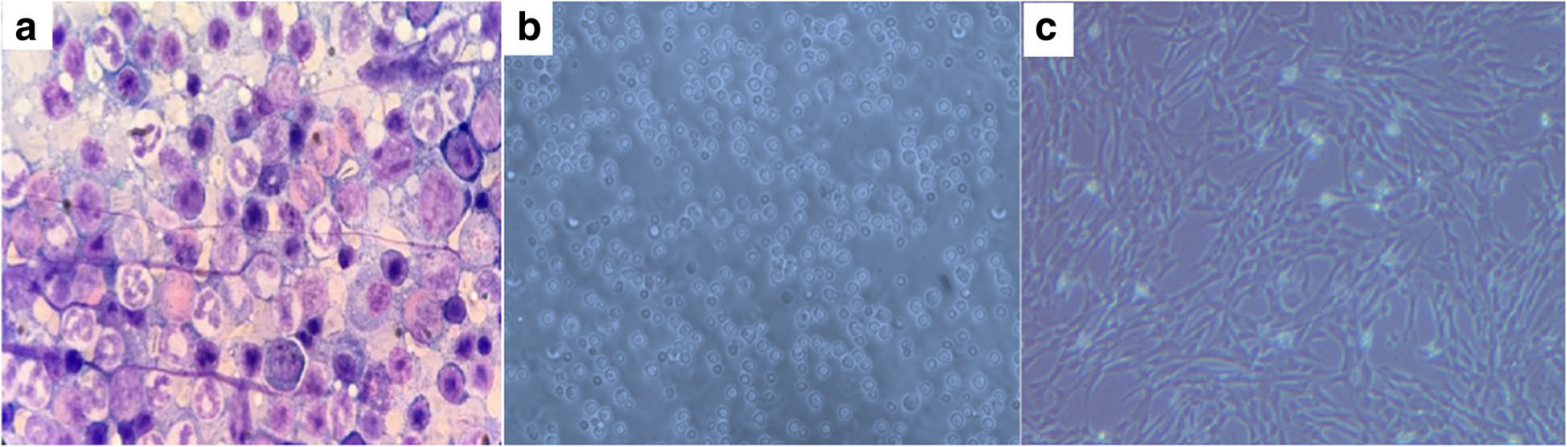
Cellular elements of rat bone marrow aspirates stained with Giemsa, × 100 (**a**). Isolated MNCs by light microscope, × 40 (**b**) and spindle shape BM-MSCs by inverted microscope, × 40 (**c**). MNCs: mononuclear cells, BM-MSCs: bone marrow mesenchymal stem cells

Stained BM with Giemsa showed different cellularity (Fig. 3a). MNCs of rats were isolated from BM (Fig. 3b) followed by adhesion after seeding on the bottom of the culture dishes. After one to 2 days from initial seeding, cells were observed by inverted microscope and it began to form extensions and some cells became spindle and appeared as fibroblast. Cells continued to proliferate and propagate until required 80% confluence and the dish area became covered with cells after 3 weeks (Fig. 3c). During expansion cultures in P2 and P3, the number of the cells kept growing. Following confluence, the cells were passed successfully up to the 10 passages (P 10). Subcultures from confluent cells tended to exhibit an accelerated growth, so the cultures reached confluence in shorter time than primary cultures. The cells started to compress and gradually lost their spindle-like appearance after over required confluence (100%).

### Counting and viability

**Fig. 4:**
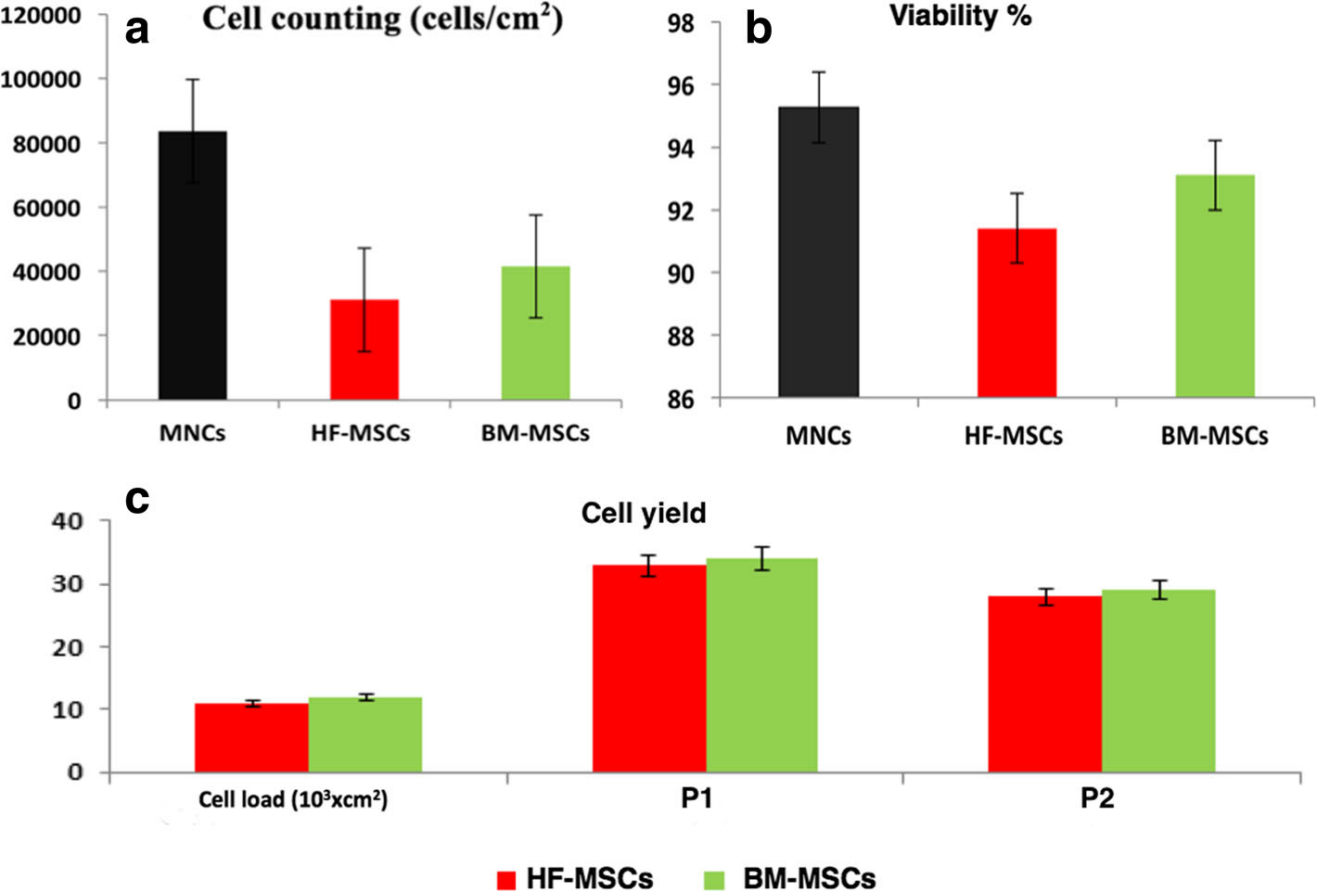
Cell counting (**a**), viability (%) (**b**), and cell yield (**c**) in the successive two passages of cultured HF-MSCs and BM-MSCs. Error bars represent Mean ± SE. MNCs: mononuclear cells, BM-MSCs: bone marrow mesenchymal stem cells, HF-MSCs: hair follicle mesenchymal stem cells, P1: passage1, P2: passage2

MNCs showed viability % of 95.27 ± 4.38 with initial counting of 8.36 × 10^4^ cells. Once HF-MSCs and BM-MSCs reached 80% confluence, the cell counting was 3.12 × 10^4^ and 4.15 × 10^4^ cells/cm^2^ while viability % was 91.41 ± 2.98 and 93.11 ± 3.06 respectively (Fig. 4a-b).

### Cell yield

The HF-MSCs and BM-MSCs yields in the successive two passages of culture were similar for both types of cells (Fig. 4c). Starting from nearly the same cell loading, the average yield was 33.15 ± 2.76 and 34.22 ± 3.99 for P1 and 28.76 ± 1.01 and 29.56 ± 3.11 for P2 respectively.

### Phenotypic analysis

**Fig. 5:**
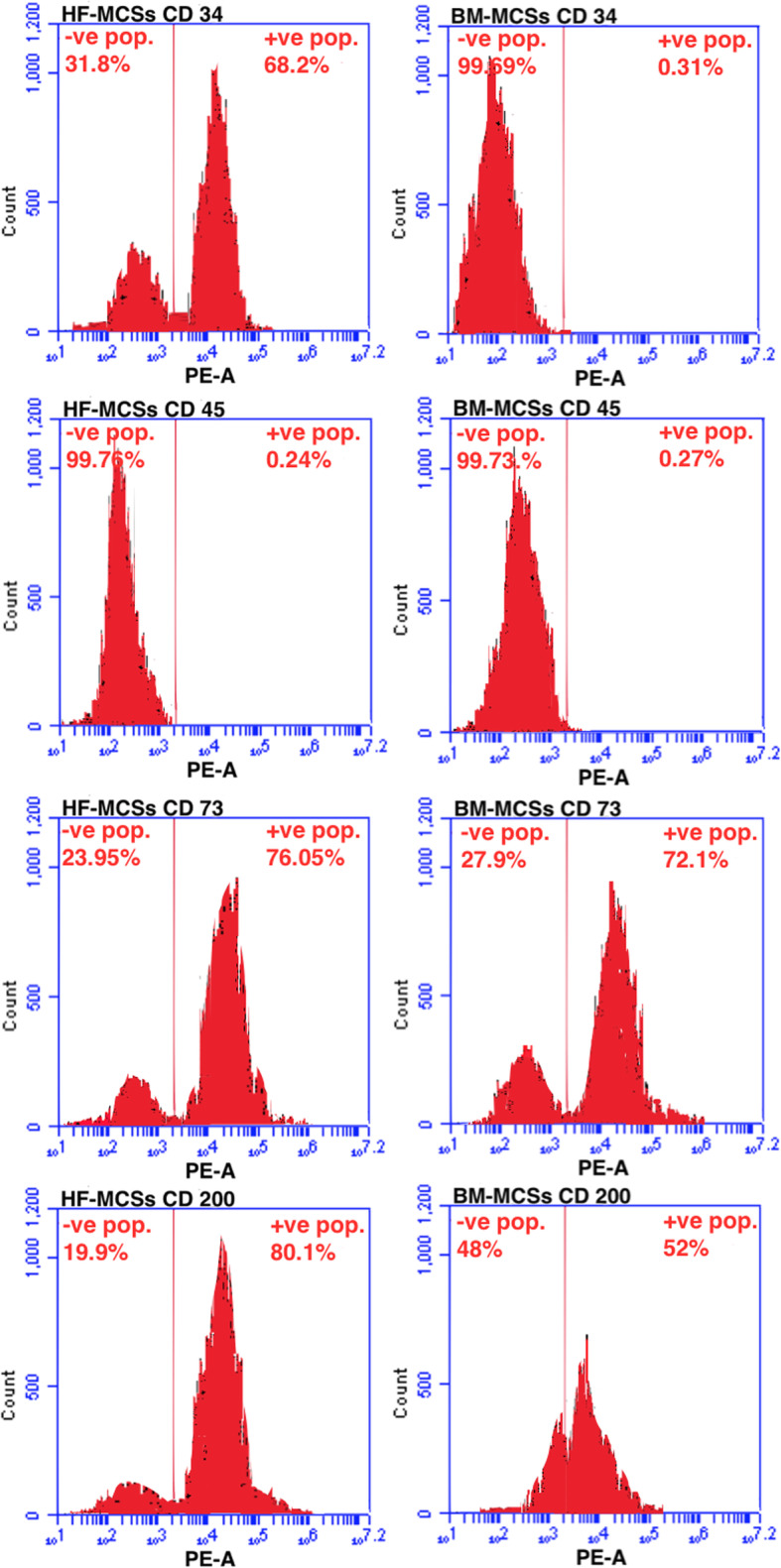
Flow cytometric analyses of HF-MSCs and BM-MSCs markers. Percentages indicate the number of cells that stained positive. HF-MSCs positively expressed CD34 (68.2%), CD73 (76.05%) and CD200 (80.1%), and negatively expressed CD45 (0.24%). BM-MSCs positively expressed CD73 (72.1%) and CD200 (52%), and negatively expressed CD34 (0.31%) and CD45 (0.27%). BM-MSCs: bone marrow mesenchymal stem cells, HF-MSCs: hair follicle mesenchymal stem cells

By flow cytometry, cells expressed MSC markers (Fig. 5). Particularly, HF-MSCs positively up-regulated CD34 (68.2%), CD73 (76.05%) and CD200 (80.1%) and down-regulated CD45 (0.24%). BM-MSCs positively up-regulated CD73 (72.1%) and CD200 (52%), however, negatively down-regulated CD34 (0.31%) and CD45 (0.27%).

### Proliferation capability

**Fig. 6:**
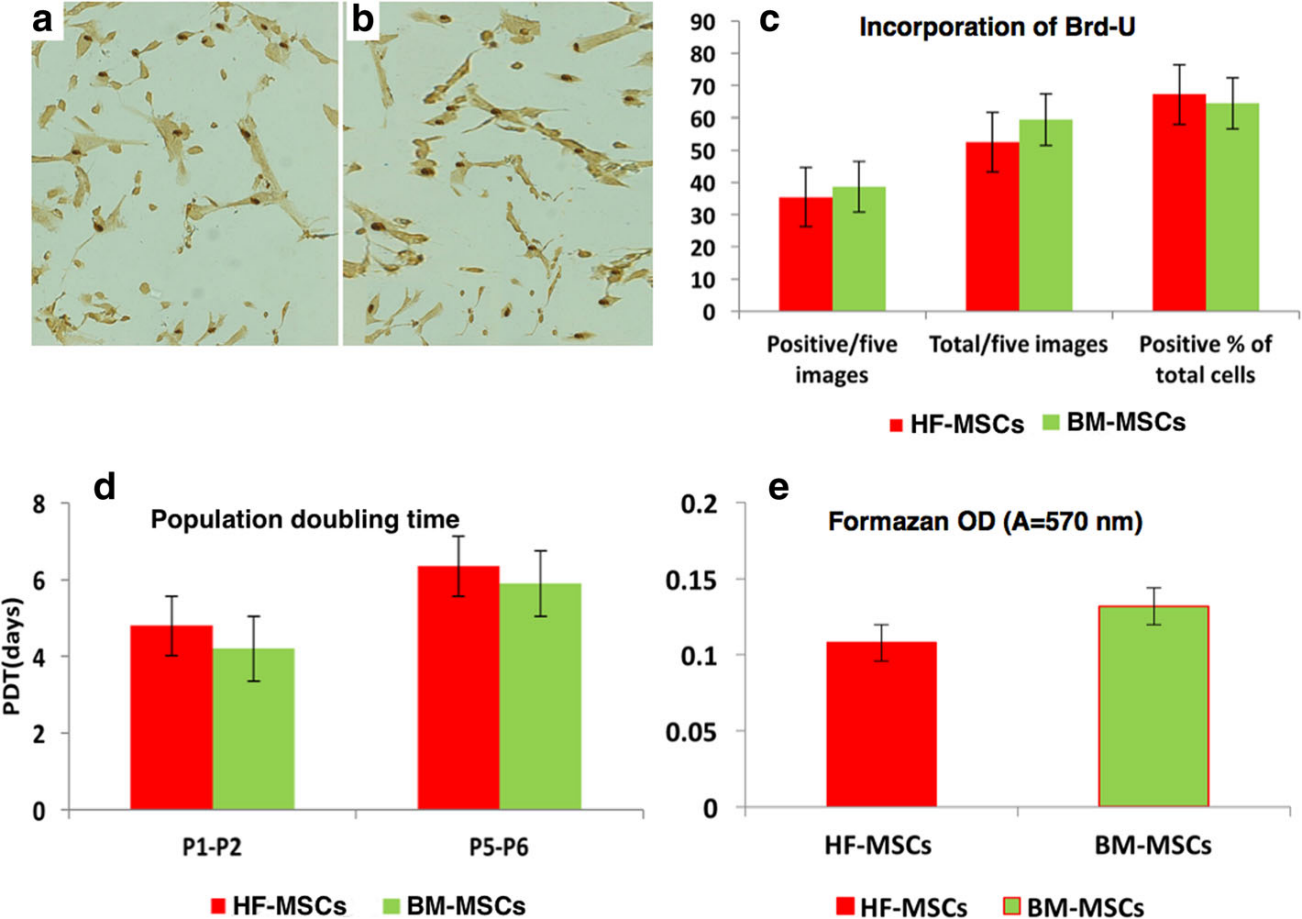
The proliferation rates of HF-MSCs (**a**) and BM-MSCs (**b**) as assessed by Brd-U uptake, × 20. The rate of Brd-U uptake was relatively similar in both cells as indicated by percentage of positive cells (67.25 ± 4.05 and 64.48 ± 3.37) in HF-MSCs and BM-MSCs respectively (**c**). The average PDT of P1 to P2 was 4.8 ± 1.3 days for HF-MSCs and 4.2 ± 0.9 days for BM-MSCs (**d**). The proliferation as expressed by OD of formazan assay of HF-MSCs was 0.108 ± 0.022 and BM-MSCs was 0.132 ± 0.009 (**e**). Error bars represent Mean ± SE. PDT: population doubling time, Brd-U: bromodeoxyuridine. BM-MSCs: bone marrow mesenchymal stem cells, HF-MSCs: hair follicle mesenchymal stem cells, P: passage

The proliferation of HF-MSCs and BM-MSCs was determined by means of incorporation of Brd-U, PDT assays and quantity of formazan release. The percentage of Brd-U positive cells in both HF-MSCs (67.25 ± 4.05) and BM-MSCs (64.48 ± 3.37) was relatively similar (Fig. 6a, b, c). The PDT was similar for both cells. The average PDT of P1 to P2 was 4.8 ± 1.3 days for HF-MSCs and 4.2 ± 0.9 days for BM-MSCs. The average PDT of P5 to P6 was 6.35 ± 2.0 days and 5.9 ± 1.4 days for HF-MSCs and BM-MSCs, respectively (Fig. 6d). The proliferation as expressed by OD of formazan assay in confluent cells (80%) of HF-MSCs and BM-MSCs was revealed that quantity of formazan released by BM-MSCs was slightly higher 0.132 ± 0.009 than HF-MSCs which was 0.108 ± 0.022 (Fig. 6e).

### In vitro adipogenic differentiation

**Fig. 7:**
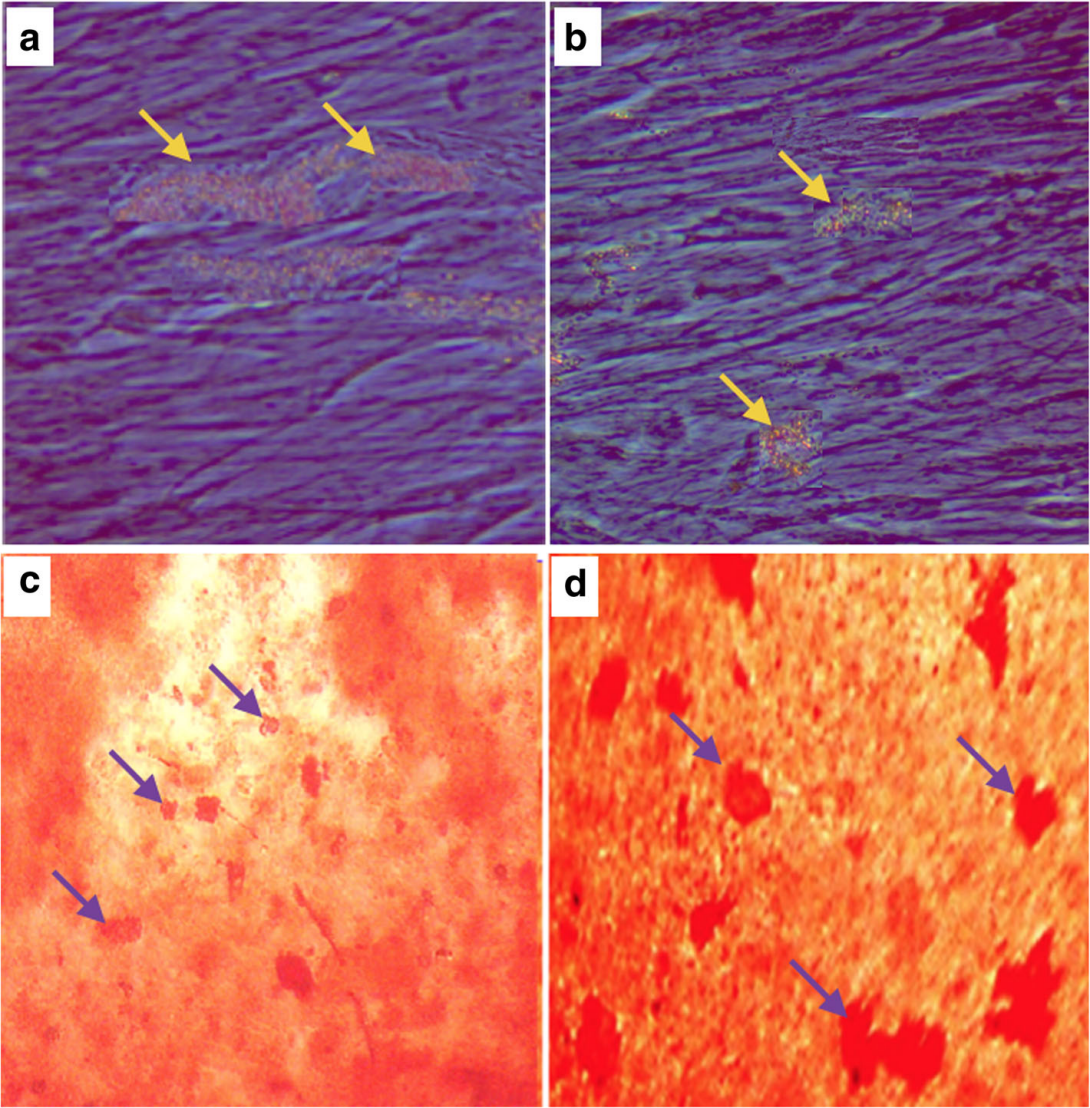
Photomicrograph of in vitro adipogenic and osteogenic differentiation of HF-MSCs and BM-MSCs. The adipogenic differentiated cells of HF-MSCs (**a**) and BM-MSCs (**b**) accumulated high and moderate oil red-O stained lipid droplets (yellow arrows) respectively (× 40). Photomicrograph of in vitro osteogenic differentiation of HF-MSCs (**c**) and BM-MSCs (**d**) showed highly and moderate scattered red calcified nodules (violet arrows) in response to Alizarin red staining respectively (× 40). BM-MSCs: bone marrow mesenchymal stem cells, HF-MSCs: hair follicles mesenchymal stem cells

Photomicrograph of in vitro adipogenic differentiation of both HF-MSCs and BM-MSCs was shown in (Fig. 7a-b). The differentiated cells of HF-MSCs showed high stain of oil red-O stained lipid droplets when compared to the BM-MSCs that showed moderate stain. Quantification of lipid accumulation by elution of oil red-O from the stained cells was illustrated as quantitative total lipid concentration values. The results showed that total lipid concentration was significantly higher (*P* < 0.05) in differentiated cells of HF-MSCs than that of BM-MSCs (Table 1).

**Table 1:** Oil concentration of in vitro adipogenic differentiation, bone alkaline phosphatase (B-ALP) activity and calcium deposition of in vitro osteogenic differentiation of HF-MSCs and BM-MSCs

Parameters	HF-MSCs	BM-MSCs
**Oil concentration (ug/10ul)**	9.788 ± 1.045*	5.402 ± 1.672
**B-ALP activity (U/mg protein)**	38.72 ± 1.54	63.45 ± 2.55**
**Calcium concentration (mg/well)**	17.88 ± 2.11	28.47 ± 3.85*

Data are represented as the mean ± SE (*n* = 5)

Asterisks * and**in the same parameter indicate significant at *P* < 0.05 and *P* < 0.01 respectively

### In vitro osteogenic differentiation

In vitro osteogenic differentiation of HF-MSCs and BM-MSCs in response to osteogenic media and stained with Alizarin red were shown in (Fig. 7c-d). Scattered red nodule like structures dispersed within the culture cells started to appear and it was seen in both cells. At day 21, photomicrograph of HF-MSCs showed low to medium scattered orange to red calcified nodules while photomicrograph of BM-MSCs showed high red calcified nodules. B-ALP activity as expressed by U/mg protein and calcium deposition (mg/well) were shown in Table 1. It was found that the activities of B-ALP and calcium deposition were significantly higher in cells of BM-MSCs than that of HF-MSCs (*P* < 0.01 and *P* < 0.05) respectively.

## Discussion

Clinical applications of cell therapy under different conditions need a multiplicity of adult stem cell sources. Although there are some models, the examination and use of adult sources are still in the infancy. There are some motionless difficulties facing scientists in keeping adult stem cells growing and reproducing in vitro while handling them to discriminate into the required needed cells. The main issue was a limited number of adult stem cells inside the body (Shi et al. 2016). Also, the unavailability and mystery of the niche where the stem cells are resided, and variety of amplification of cell in vitro with the maintenance of cell regenerative capability (Maruyama et al. 2015). The problem associated with the process of adult stem cell transplantation for regeneration is the possibility of tumors risk. Therefore, it is important to induce the stem cells for required functional cells before transplantation, which is the prospect study of adult stem cells (Bu et al. 2017).

Each source of adult stem cells has many advantages and disadvantages that were described previously (Oryan et al. 2017). Benefits of BM source are a massive number of cells harvested, easily collected, and stable cells during seeding. The disadvantages are euthanization, the pain and the risk of infection during the collection process besides the little proliferation ability. The advantage of HF-MSCs was the easily collection without ethics, no epigenetic handling, and non-carcinogenic (Hoffman and Amoh 2018). In this work, characteristic of adult stem cells from HFs (HF-MSCs) as another source was studied to be used as an alternative source. Commonly, the three essential features allowing MSCs to be purified from different homes are the ability to stick to plastic surfaces, the capability for proliferation and the susceptibility to trypsin digestion (Oryan et al. 2017). Generally, MSCs are harvested from different sources and their multipotent property enables them to potentially differentiate into cells of several tissues such as bone, ligament, liver cells, fibroblasts and adipocytes (Brevini and Gandolfi 2013). Up to date, little is available on the comparative isolation, purification, characterization, proliferation, rapid amplification and differentiation of rat MSCs derived from the HF and BM from the same animal. This work hopes to use HF-MSCs as an additional mature stem cell source for therapy.

When HF-MSCs and BM-MSCs reached 80% confluence, the cell counting and viability % were nearly similar. The cell yields in the successive two passages of culture were identical for both types of cells. Higher yield at harvest of HF-MSCs was previously recorded (Hoogduijn et al. 2006). The proliferation of HF-MSCs and BM-MSCs was determined by means of the quantity of formazan release, incorporation of Brd-U, and PDT assays. The percentage of Brd-U positive cells and PDT were relatively similar in both types of cells. The proliferation, as expressed by OD of formazan assay in confluent cells revealed that the quantity of release by BM-MSCs was slightly higher than HF-MSCs.

Clusters of differentiation (CDs) analysis revealed that HF-MSCs were positively up-regulated expressed CD34, CD73 and CD200, and negatively expressed CD45. BM-MSCs positively up-regulated CD73 and CD200; however, down-regulated expressed CD34 and CD45. These data are parallel to the previous work of Hoogduijn et al. (Hoogduijn et al. 2006) who reported that the majority of the HF-MSCs derived from the dermal layer of rat skin expressed the mesenchyme markers CD44, CD73, CD90, CD29 and CD105 and this expression configurations were identical comparable to MSCs isolated from BM. The only disagreement was in the expression pattern of two CDs, the first was CD34, who reported that HF-MSCs did not express it as BM-MSCs and the second was the degree of expression of CD73, as it was significantly lower in BM-MSCs than in HF-MSCs. In the current study, the expression of CD73 by HF-MSCs was slightly higher (76.05%) than BM-MSCs (72.1%). The expression of surface protein CD34 as a marker for mouse HF-MSCs reside in the bulge region was first mentioned previously (Trempus et al. 2003). Essentially, the CD34-positive cells can be easily isolated from the follicles of the mouse skin. Therefore, CD34 is not considered as a hematopoietic stem cell marker in the mouse (Osawa et al. 1996) as in humans (Cotsarelis 2006). However, CD34 denotes the ideal marker for rodent HF-MSCs and affords a respected item for discovering bulge cell behaviors (Cotsarelis 2006). HF-MSCs CD34 may be to refresh hematopoietic system, as mentioned previously (Lako et al. 2002). Some studies were focused on the surface marker of HF-MSCs such as integrin, keratin (CK14, CK15, and CK19), C8/144B, p63, CD71 and CX43 (Dong et al. 2014; Maleki et al. 2014; Son et al. 2015), CD34 (Sol et al. 2018) CD49f (Krebsbach and Villa-Diaz 2017) and CD34 and CD49f duel-positive cell residents in mice (Fontenete and Perez-Moreno 2018). Babakhani et al. (Babakhani et al. 2019) showed that the bulge of HF-MSCs was nestin and CD34 positive and Kr15 negative.

Previous scientists were candidate HF-MSCs in the future as very suitable stem cells due to some issues for example, rapid availability, easy culture, high proliferative potential and differentiated to many cell types (Gilanchi et al. 2014) as keratinocytes (Saeidinia et al. 2017) and endothelial cells (Xu et al. 2014). Furthermore, it can be used in the enhancement of wound healing (Heidari et al. 2016), growth of nerve supply to HF (Hoffman and Amoh 2018), and degenerative spinal nerve repair (Amoh et al. 2012). HF-MSCs are therefore considered as a perfect cell source for cell therapy. The question of to what extent the work in rat or mice HF is a benefit for human HF is conflicting. The current study used large whisker HFs from rats that are structurally identical to human HFs. There was a difference in isolated rat and human MSCs. Besides, differences were documented in rat and human BM-MSCs, and the resemblances in this work between rat BM-MSCs and HF-MSCs, it is likely that human HF-MSCs will perform more equally to human BM-MSCs than to rat HF-MSCs.

Adipogenic differentiation of both HF-MSCs and BM-MSCs was studied in this work. The differentiated BM-MSCs showed moderate accumulation of oil red-O stained lipid droplets when compared to the HF-MSCs that exhibited high stain. Quantification of lipid accumulation by elution of oil red-O from the stained cells was illustrated as quantitative total lipid concentration values. The results exposed that total lipid concentration was significantly higher in HF-MSCs than BM-MSCs. It is known that there was a cross-talk between HF-MSCs in the HF and the surrounding adipose tissue. It was documented that both adipocytes of adipose tissue and HF were undergoing a parallel increase in growth, proliferation, and thickness (Plikus et al. 2008; Rompolas et al. 2013). The secretion of one of them can affect the behavior of other in the same direction; for example, adipocytes secrete bone morphogenic protein-2 (BMP2) during the phases of late down-regulation and early resting, which help the latent condition in the bulge region, while release of BMP2 was lowered at the end of the resting phase with the stimulation of HF-MSCs (Yi 2017). Alterations of delaying the hair growth have been found to prevent adipogenesis, which suggested previously that HF-MSCs direct signals for activation of adipogenesis (Turksen 2015). Under normal conditions, the growth phase of the HFs was extended by the adipose tissue around it or subcutaneously (Zhang et al. 2014; Nilforoushzadeh et al. 2017). Proliferation and multiplication of adipocytes have been detected during the conversion of the HF from the resting to the growth phase (Festa et al. 2011; Huang et al. 2016). The parallels of a defect in both adipocyte or lipid metabolism disorders and HFs were documented. Zhang et al. (Zhang et al. 2014) reported that hyperlipidemia in transgenic mice was accompanied by overexpression of apolipoprotein C1 (APOC1) and hair growth disorders. We concluded that elevated adipogenesis of HF-MSCs may be due to the important function of skin adipose tissue, including energy homeostasis, thermogenesis, endocrine capacity and immune modulatory effect.

Staining of in vitro osteogenic differentiation of HF-MSCs and BM-MSCs by Alizarin red after 21 days revealed that HF-MSCs showed low to medium scattered orange to red calcified nodules. In contrast, the photomicrograph of BM-MSCs showed high red calcified nodules. B-ALP activity and calcium deposition were significantly higher in BM-MSCs than that of HF-MSCs. These data indicated the opposite picture of adipogenic differentiation, a data agreed with Schug et al. (Schug et al. 2011), who mentioned that elevated adipogenic differentiation might result in a lowered sum of stem cells obligating to the osteoblastic lineage and might reduce the capability of stem cells to undergo osteogenic differentiation. Also, several factors have been discussed in the literature to inhibit osteogenesis and on the same way, stimulated adipogenesis and vice- versa (Atashi et al. 2015; Watt and Schlezinger 2015). In bone, BM-MSCs differentiate into osteoblasts and adipocytes with an inverse relationship. When the BM-MSCs differentiate into osteoblasts, the adipogenic differentiation is weakened; when the BM-MSCs differentiate into adipocytes, the osteogenic differentiation is weakened (Yeung et al. 2005). Thompson et al. (Thompson et al. 1998) indicated that BM-MSCs could differentiate into osteoblasts and fat cells under natural conditions without any intervention, both of which maintain a dynamic equilibrium. If the balance is disrupted, a metabolic bone disease such as osteoporosis will occur. The relationship between bone formation and adipogenesis is complicated in the bone marrow microenvironment. Adipogenesis and osteogenesis of MSCs differentiation are reciprocally regulated processes through various active secretion of osteogenic and adipogenic mediators (Chen et al. 2016).

The present findings demonstrate that the HF-MSCs are very similar in most tested characteristics to BM-MSCs with the exception of osteogenic and adipogenic fate. Additionally, no issues have been reported during the collection of HF-MSCs. Therefore, the HF may represent a suitable and accessible source for adult stem cells and can be considered an ideal cell source for adipogenesis research.

## Data Availability

Please contact authors for data requests.
